# Failed internal fixation due to osteonecrosis following traumatic periprosthetic fracture after hip resurfacing arthroplasty: A case report and review of the literature

**DOI:** 10.3109/17453670903413152

**Published:** 2009-12-04

**Authors:** Jozef Zustin, Eugen Winter

**Affiliations:** ^1^Institute of Pathology, University Medical Centre Hamburg Eppendorf, Hamburg; ^2^Trauma Surgery, Orthopedics and Arthroplasty Clinic, Klinikum Friedrichshafen, Friedrichshafen, Germany

## Introduction

A 55-year-old man had undergone a Birmingham Hip Resurfacing (Cup 56, Head 50; BHR; Smith and Nephew TLC, London, UK) for primary osteoarthritis. The index procedure and postoperative healing were uneventful and radiographs showed well-fixed femoral and acetabular components.

**Figure 1.**
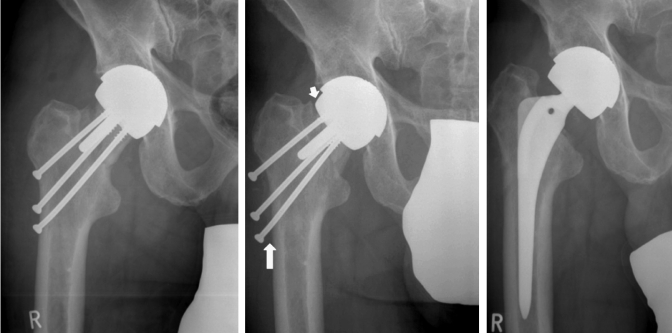
A. Traumatic periprosthetic femoral neck fracture 18 weeks after the index procedure treated with cannulated cancellous screws. B. Collapse of the femoral neck (short arrow) led to marked dislocation of both the proximal femoral remnant and all 3 screws (long arrow). C. After the revision surgery with conversion to a femoral stemmed total hip replacement with extra-large femoral head.

18 weeks after surgery, the patient was involved in a motorcycle accident and fell on the operated hip. An undislocated vertical fracture of the femoral neck was treated surgically to preserve the prosthesis. As the centrally located implant stem might cause difficulty with the placing of typical implants (e.g. screw-plate device or a cephalomedullary nail), 3 parallel cannulated cancellous screws in a triangular configuration (Figure 1A) were used for fixation of the fracture. Care was taken to avoid contact between the implanted stainless steel screws and both the stem and lateral walls of the femoral component. The patient was operated on 14 h after the injury.

11 weeks later, the patient presented with hip pain. Radiographs showed 15-mm descent of the femoral component and a dislocation of all 3 screws (Figure 1B). The arthroplasty was converted to a femoral stemmed total hip replacement with extra-large femoral head (cement-free BiCONTACT System, Aesculap AG, Tuttlingen, Germany) (Figure 1C) and retention of the well-fixed acetabular component. Follow-up at 8 months was uneventful.

Retrieved femoral remnant tissue with the in situ femoral component and several bone tissue fragments from the neck obtained at revision surgery were immediately fixed in buffered formalin for further analysis. The specimen was cut with the femoral component in situ, by a diamond-coated band saw, into 4 quadrants and analyzed macroscopically and by contact radiography (Faxitron X-Ray LLC, Wheeling, IL). Both the medial and lateral sections of the central slice were completely embedded in plastic after cement dissolution with acetone and removal of the prosthesis. The specimens were processed undecalcified and embedded in methylmethacrylate. From each undecalcified processed plastic block, one 5-μm section was cut with a heavy-duty microtome. The sections were stained by the toluidine blue staining method.

**Figure 2.**
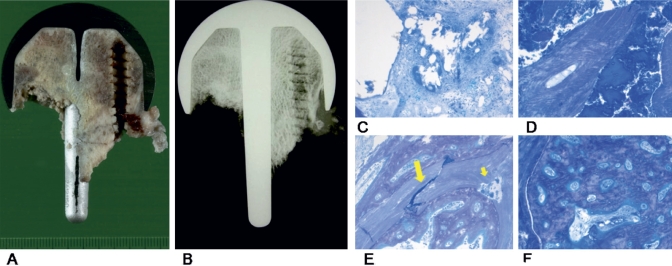
A. Well-fixed femoral component, narrow superficial zone of cement penetration, and irregular fracture line were apparent macroscopically on the cut plane. B. Absence of vital reaction to the screw was evident by contact radiography several weeks after the procedure. C. Cement granulomas and fibrosis were found at the bone-cement interface. D. Bone trabeculae without stainable osteocytes and precipitations of calcium salts were found in the vicinity of the screw and of the fracture line. E. Bone tissue fragments from femoral neck distally to the fracture line revealed several fractured individual bone trabeculae (long arrow) with formation of microcallus and focal resorptive changes (short arrow). F. Broad fracture callus within the isolated bone fragments from femoral neck distal to the fracture line.

Macroscopically (Figure 2A), a well-fixed femoral component with minimal cement mantle and focal superficial cement penetration was found. Contact radiography of the specimen revealed no reaction to the intraosseously located screw (Figure 2B).

Microscopically, foreign body granulomas and loose fibrosis were apparent at the bone-cement interface (Figure 2C). The remaining bone tissue showed a loss of stainable osteocytes and disorganized intertrabecular bone marrow. More distally, many dead, irregular bone fragments—so-called “bone chips” (Doorn et al. 1996)—without resorptive reaction were found in the vicinity of both the intraosseous screw and the fracture line (Figure 2D). In contrast with the former, multifocal fracture of bone trabeculae associated with the formation of microcallus (Figure 2E) and broad areas of mineralized callus (Figure 2F) were present in the bone fragments removed from the rest of the femoral neck at the revision surgery. We found no fibrocartilaginous tissue, characteristic of pseudoarthrosis.

## Discussion

Hip resurfacing arthroplasty is currently the fastest growing hip procedure worldwide (Amstutz and Le Duff 2008). One of the major complications associated with resurfacing is periprosthetic femoral neck fracture (Shimmin and Back 2005). As this is not usually associated with trauma, change in biomechanical properties and/or pathological alterations within the femoral remnant bone tissue are believed to be causative. Typical non-traumatic periprosthetic fractures are usually treated with revision to a stemmed femoral component, although there have been several reports of successful nonoperative management (Cumming and Fordyce 2003, Cossey et al. 2005, Morgan et al. 2008).

With wide use of the hip resurfacing procedure in young active patients, it is likely that the number of traumatic periprosthetic fractures will increase. Apart from revision surgery with conversion to total hip arthroplasty, several alternative operative and nonoperative therapeutic options might be considered. Recently, several reports of successful operative treatment of traumatic periprosthetic fractures after hip resurfacing have been published (Aning et al. 2005, Mereddy et al. 2008, Weinrauch and Krikler 2008, Orpen et al. 2009, Whittingham-Jones et al. 2008) (See Table).

**Table:** Overview of successful operative treatment of traumatic periprosthetic fracture after hip resurfacing arthroplasty

Age	Sex	Preoperative diagnosis	Time to fracture	Trauma	Fracture type	Therapy	Follow-up	Reference
32	F	posttraumatic arthritis	4 years	car accident	comminuted sub-trochanteric	contoured broad AO DCP	5 months	Whittingham-Jones (2008)
54	M	osteoarthritis	2 years	–	displaced inter-trochanteric	locking plate 3 screws	1 year	Orpen (2009)
54	M	osteoarthritis	3 months	fall from a ladder	multi-fragmented reverse oblique intertrochanteric	locking plate 3 screws	6 months	Orpen (2009)
67	M	osteoarthritis	19 months	fall on a hip	closed intertrochanteric	130°angled blade plate	17 months	Weinrauch (2008)
69	M	osteoarthritis	15 months	fall	undisplaced basicervical	3 cannulated cancellous screws	4 years	Mereddy (2008)
60	M	–	2 years	traffic accident	complex multifragmentary	2 locking screws cerclage wires	1 year	Aning (2005)

As vital reaction to cement was apparent histologically at the bone-implant interface in our case, we believe that the femoral bone remnant was viable after implantation of the hip resurfacing arthroplasty. The absence of vital reactions to the screw used for the fracture fixation and resorptive as well as reparative changes proximal to the fracture line were, however, suggestive of osteonecrosis. The other 2 screws were implanted somewhat more distally, and ended within the collapsed necrotic bone tissue. Loss of stainable osteocytes in the bone trabeculae was apparent throughout the remnant of the femoral head. The remaining vital femoral neck fragments showed vital mineralized fracture callus formations.

As trauma-induced avascular necrosis of the femoral head, either with or without fracture, represents the most common form of aseptic femoral head necrosis (Bachiller et al. 2002), we suggest that posttraumatic osteonecrosis of the femoral remnant caused the treatment failure of the periprosthetic fracture in our patient. This led to the collapse of necrotic bone tissue several weeks after the operative treatment, followed by descent of the prosthesis and the screws used for the fracture fixation.

Periprosthetic fracture after hip resurfacing replacement is a well-documented early-onset complication of this modern arthroplasty method, with an incidence of 1–3% (Beaule et al. 2004, Amstutz et al. 2004, De Smet 2005, Shimmin and Back 2005, Marker et al. 2007, Amstutz and Le Duff 2008, Steffen et al. 2008). Osteonecrosis in femoral remnants has been found in recent retrieval analyses (Little et al. 2005, Steffen et al. 2008), and was considered causative for most typical periprosthetic fractures due to weakening of the bone structure in the absence of adequate trauma. In contrast to these fractures, analysis of our single case with periprosthetic fracture following adequate trauma revealed vital reactions at the bone-cement interface after the index procedure. However, the subsequent accident led to 2 additional important events (fracture and its operative therapy) that had an adverse influence on further viability of the bone remnant tissue, which led to its failure. Thus, we suggest that traumatic fractures can take place in the absence of osteonecrosis in femoral remnant—even at later follow-up. Furthermore, we have demonstrated that osteonecrosis can appear in such cases secondary to attempts to treat the traumatic periprosthetic fracture operatively.

We believe that the risk of secondary osteonecrosis is high after operative treatment of traumatic periprosthetic fracture after resurfacing replacement; thus, a revision to total hip arthroplasty is probably preferable in such cases.
